# Investigation of Modal Characteristics of Silicon Nitride Ridge Waveguides for Enhanced Refractive Index Sensing

**DOI:** 10.3390/mi16020119

**Published:** 2025-01-21

**Authors:** Muhammad A. Butt, Lukasz Kozlowski, Mateusz Słowikowski, Marcin Juchniewicz, Dagmara Drecka, Maciej Filipiak, Michał Golas, Bartłomiej Stonio, Michal Dudek, Ryszard Piramidowicz

**Affiliations:** 1 Institute of Microelectronics and Optoelectronics, Warsaw University of Technology, Koszykowa 75, 00-662 Warsaw, Poland; 2 The Centre for Advanced Materials and Technologies CEZAMAT, Warsaw University of Technology, Poleczki 19, 02-822 Warsaw, Poland; 3 Institute of Applied Physics, Military University of Technology, Gen. Sylwestra Kaliskiego 2, 00-908 Warsaw, Poland

**Keywords:** silicon nitride, ridge waveguide, racetrack ring resonator, sensitivity enhancement, effective refractive index

## Abstract

This paper investigates the wavelength-dependent sensitivity of a ridge waveguide based on a silicon nitride (Si_3_N_4_) platform, combining numerical analysis and experimental validation. In the first part, the modal characteristics of a Si_3_N_4_ ridge waveguide are analyzed in detail, focusing on the effective refractive index (n_eff_), evanescent field ratio (EFR), and propagation losses (α_prop_). These parameters are critical for understanding the interplay of guided light with the surrounding medium and optimizing waveguide design for sensing applications. In the second part, the wavelength-dependent sensitivity of a racetrack ring resonator (RTRR) based on the Si_3_N_4_ waveguide is experimentally demonstrated. The results demonstrate a clear increase in the sensitivity of the RTRR, rising from 116.3 nm/RIU to 143.3 nm/RIU as the wavelength shifts from 1520 nm to 1600 nm. This trend provides valuable insights into the device’s enhanced performance at longer wavelengths, underscoring its potential for applications requiring high sensitivity in this spectral range.

## 1. Introduction

Silicon nitride (Si_3_N_4_) photonics is an essential platform in modern integrated photonics that is known for its versatility and exceptional optical properties [1,2]. Si_3_N_4_ offers a wide optical transparency range, extending from the visible spectrum to the MIR (400 nm to 6.7 µm) [3], making it suitable for diverse applications such as telecommunications, biosensing, quantum computing, and LiDAR [4,5,6,7]. One of its primary advantages is its ultra-low optical loss, particularly in the near-infrared region, where losses can be less than 1 dB/cm [8]. The material’s moderate refractive index (∼2.0) enables efficient mode confinement while maintaining robustness against fabrication imperfections unlike higher contrast materials such as silicon [9]. Its compatibility with CMOS fabrication processes ensures scalability and cost-efficiency, facilitating seamless integration with silicon photonics [10,11]. Si_3_N_4_ also exhibits high thermal stability and a low thermo-optic coefficient, making it ideal for temperature-sensitive applications [12]. These features collectively position Si_3_N_4_ photonics as a key enabler for next-generation photonic integrated circuits (PICs), addressing the needs of both research and industry [13,14].

Optical waveguides are physical structures that guide light waves along a specific path, and they are often used in telecommunications, sensors, and photonics [15]. They confine and direct light through reflection and refraction, typically in the form of optical fibers or planar waveguides [16]. These waveguides play a crucial role in modern technology, supporting high-speed data transmission and advanced optical systems. The sensitivity of a waveguide is intrinsically tied to its dimensions because these determine how the electromagnetic field of the guided mode interacts with the surrounding environment [17,18]. In a waveguide, the dimensions influence the mode profile, effective index (n_eff_), and evanescent field strength [19,20]. Waveguide sensitivity is significantly influenced by its cut-off wavelength, which is determined by the physical geometry and size of the waveguide [21,22]. This sensitivity to cut-off wavelength makes waveguides ideal for selective wavelength transmission but also introduces design constraints, as the waveguide must be precisely engineered to accommodate the desired wavelength range [23].

To design highly sensitive photonic sensors, it is essential to maximize the overlap of the guided mode’s electric field with the surrounding ambient medium. This enhanced overlap upsurges the interaction between the optical mode and the external environment, significantly improving the sensor’s ability to detect changes in the medium, for instance, variations in refractive index (RI), chemical concentrations, or other perturbations [24,25]. However, achieving this greater interaction often comes at the cost of increased waveguide losses. These losses can result from several factors, including radiation losses due to energy leakage into unguided modes, scattering losses caused by imperfections at the waveguide interface, and material absorption within the ambient medium [26]. In this paper, we comprehensively analyze the modal characteristics of the Si_3_N_4_ waveguide, focusing on the evanescent field ratio (EFR), propagation loss (α_prop_), and sensitivity across a broad wavelength spectrum. This investigation offers valuable insights into the relationship between sensitivity and wavelength. Furthermore, we experimentally demonstrate this phenomenon using a Si_3_N_4_-based RTRR, measuring its sensitivity across six distinct wavelength regions.

## 2. Waveguide Design and Numerical Analysis

**Figure 1:**
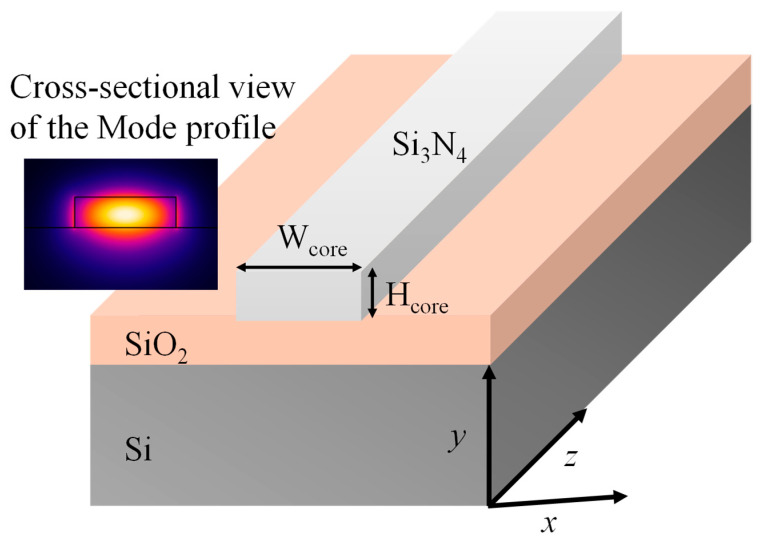
Graphic of a Si_3_N_4_ ridge waveguide. The inset shows the norm. E-field distribution at 1550 nm.

The ridge waveguide consists of a Si₃N₄ core deposited on a SiO₂ on Si substrate as shown in Figure 1. The core dimensions are characterized by their height (H_core_) and width (W_core_). The geometric parameters utilized for the waveguide analysis are detailed in Table 1. The refractive indices of Si₃N₄ and SiO₂ were sourced from the well-established works of Luke et al. [27] and Kischkat et al. [28], respectively, ensuring accurate and reliable material parameters for the analysis. COMSOL Multiphysics 5.5 is a powerful simulation software widely used for analyzing optical waveguide-based photonic devices. It provides a versatile platform to model the electromagnetic behavior of photonic structures by solving Maxwell’s equations with high accuracy. By employing the Wave Optics Module, we analyzed critical parameters such as mode profiles, effective refractive indices (n_eff_), propagation losses (α_prop_), and wavelength-dependent behavior [29].

**Figure 2:**
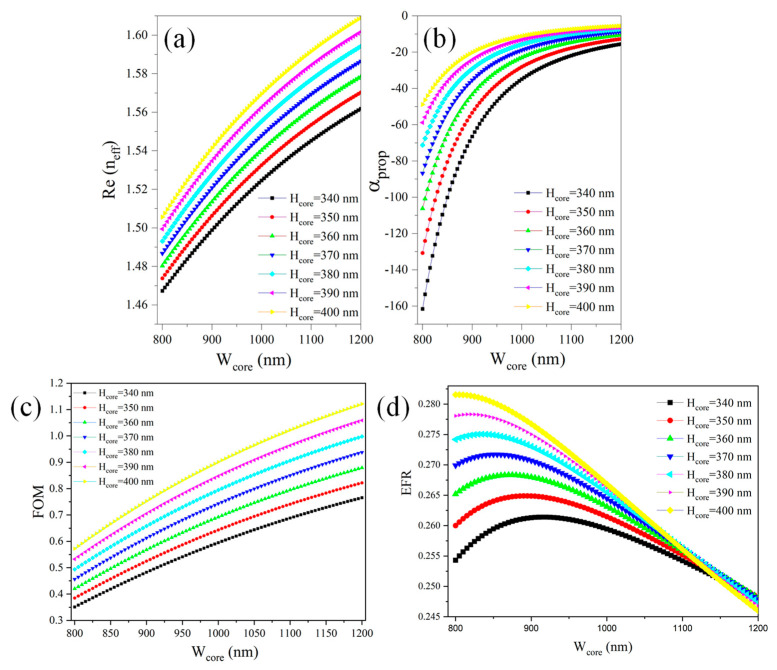
(**a**) Re(n_eff_) versus waveguide dimensions, (**b**) α_prop_ versus waveguide dimensions, (**c**) FOM versus waveguide dimensions, and (**d**) EFR versus waveguide dimensions,.

At first, we employed boundary mode analysis in COMSOL Multiphysics 5.5 software to determine the n_eff_ of a waveguide, which can have both real (Re) and imaginary (Im) components. This analysis involves solving the electromagnetic (EM) field equations at the boundaries of the waveguide cross-section to identify the supported modes and their corresponding effective refractive indices. By defining the waveguide’s geometry, material properties, and operating wavelength, COMSOL calculates the distribution of the electric and magnetic fields in the waveguide. The Re(n_eff_) represents the phase velocity of the propagating mode relative to the speed of light in a vacuum and determines the wave’s propagation characteristics within the waveguide. Figure 2a reveals that as the core size increases, Re(n_eff_) rises, indicating stronger light confinement within the waveguide core.

The Im(n_eff_), on the other hand, quantifies the attenuation of the mode as it propagates, indicating losses due to absorption, scattering, or leakage. The α_prop_ measured in dB/cm is linked to Im(n_eff_) through the following Equation (1). The accurate determination of both components is crucial for designing efficient photonic devices, as the real part ensures proper confinement and guidance of light, while the imaginary part provides insights into energy losses.

(1) $$\alpha \left(\frac{dB}{cm}\right)=\left(\frac{4\times \pi \times Im(n_{eff})}{\lambda }\right)\times 4.343;$$

Here, λ represents the wavelength of the propagating light. This equation demonstrates that a higher Im(n_eff_) leads to increased attenuation per unit length. Figure 2b shows that as the waveguide dimensions increase, α_prop_ decreases, leading to a reduction in mode leakage to the substrate. This is further corroborated by the waveguide’s figure of merit (FOM), which indicates enhanced performance and improved confinement as the dimensions grow.

The ratio of mode confinement in the core to power leakage into the substrate can be considered an FOM for evaluating the performance of an optical waveguide, especially in contexts where guiding efficiency and minimizing losses are critical (Figure 2c). It is determined by performing a surface integral of the electric field intensity squared over the core region and dividing it by the surface integral of the electric field intensity squared over the substrate region. In this case, the FOM would quantify how effectively the waveguide confines the optical mode within the core relative to the unwanted leakage into the substrate. Mathematically, it could be expressed in the form of Equation (2):

(2) $$FOM=\frac{Modeconfinementincore}{Modeleakagetosubstrate}=\frac{\int_{core}E^{2}dA}{\int_{substrate}E^{2}dA};$$

The FOM of the Si₃N₄ ridge waveguide improves as the waveguide dimensions increase. This enhancement is primarily attributed to a reduction in mode power leakage to the substrate and a decrease in the evanescent field. These factors, in turn, lower the Evanescent Field Ratio (EFR) of the waveguide, leading to a decrease in the device’s sensitivity. The EFR quantifies the relative strength of the evanescent field compared to the total field within an optical waveguide [30]. For a ridge waveguide, the EFR is defined as the ratio of the power or intensity of the evanescent field in the upper cladding to the total field intensity, which includes the contributions from the substrate, upper cladding, and core. The EFR is expressed in the form of Equation (3).

(3) $$EFR=\frac{\iint_{uppercladding}{\left|E(x,y)\right|}^{2}dxdy}{\iint_{total}{\left|E(x,y)\right|}^{2}dxdy};$$

**Figure 3:**
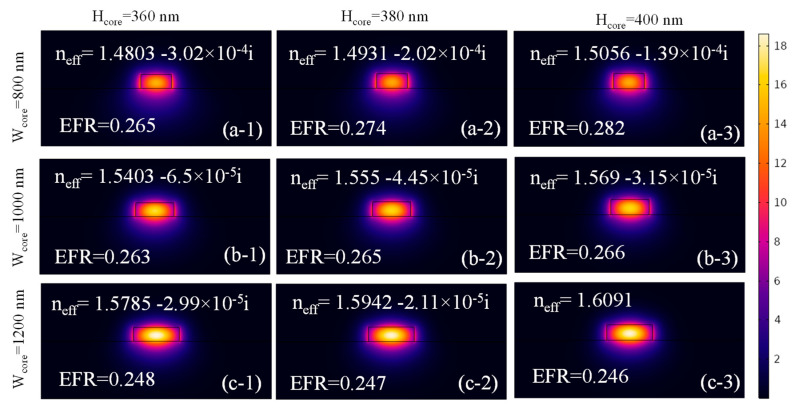
Norm. E-field distribution in the Si_3_N_4_ ridge waveguide at an operational wavelength = 1550 nm for (**a-1**,**b-1,c-1**) H_core_ = 360 nm and W_core_ = 800 nm, (**a-2**,**b-2**,**c-2**) H_core_ = 380 nm and W_core_ = 1000 nm, and (**a-3**,**b-3**,**c-3**) H_core_ = 400 nm and W_core_ = 1200 nm.

Figure 3(a-1)–(c-3) illustrates the normalized E-field distribution in the Si₃N₄ ridge waveguide for core dimensions ranging from W_core_ = 800 nm to 1200 nm and H_core_ = 360 nm to 400 nm. As the waveguide dimensions increase, Re(n_eff_) rises due to better mode confinement, while Im(n_eff_) decreases. This reduces power leakage to the substrate and minimizes the evanescent field surrounding the waveguide core. For W_core_ = 800 nm and H_core_ = 400 nm, the EFR is 0.282, which decreases to 0.246 when the dimensions are expanded to W_core_ = 1200 nm and H_core_ = 400 nm. These results highlight the importance of optimizing waveguide geometry to achieve superior light confinement and minimal propagation losses.

**Figure 4:**
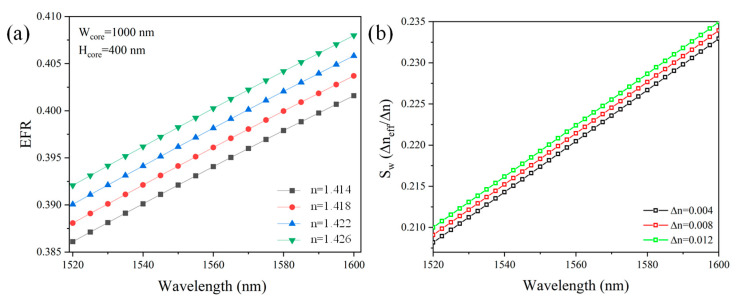
(**a**) EFR versus wavelength, (**b**) S_w_ versus wavelength. Note: In this analysis, W_core_ and H_core_ are maintained at 1000 nm and 400 nm, respectively.

For sensors operating based on the wavelength interrogation method, it is crucial to evaluate both the EFR and the sensitivity of the waveguide (S_w_) across a broad wavelength spectrum. For this analysis, the waveguide dimensions are fixed at W_core_ = 1000 nm and H_core_ = 400 nm. This selection is based on the specific design parameters of the RTRR, which is fabricated and analyzed in detail in the subsequent section. These dimensions were selected to align with the experimental setup, allowing for a clear and accurate comparison between theoretical predictions and experimental results. The EFR of the waveguide over a wavelength range from 1520 nm to 1600 nm was analyzed, as depicted in Figure 4a. The EFR of the waveguide increases linearly with the operational wavelength. The RI measurement range of 1.414 to 1.426 was chosen for presentation in numerical and experimental studies because this range corresponds to the refractive indices of many bioanalytes, making it particularly relevant for applications in biological and chemical sensing. Moreover, a higher ambient RI results in a more pronounced increase in the EFR, highlighting the influence of the surrounding medium on the waveguide’s performance. This comprehensive assessment provides valuable insights into the waveguide’s performance across the spectrum.

The sensitivity of an optical waveguide quantifies the ability of the waveguide to detect changes in the RI of the surrounding medium as presented in Equation (4).

(4) $$S_{w}=\frac{∆n_{eff}}{∆n};$$

Here, Δn_eff_ represents the variation in the effective refractive index of the guided mode caused by the variation in the surrounding medium’s refractive index (∆n). A higher sensitivity indicates that even a small Δn produce a significant shift in n_eff_, enhancing the detection capabilities of the waveguide. Sensitivity counts on several factors, including the waveguide geometry, the refractive index contrast between the core and the cladding, and the degree of mode confinement. From Figure 4b, it is observed that S_w_ of the waveguide for Δn = 0.012 is approximately 0.21 at an operational wavelength of 1520 nm. This sensitivity increases progressively, reaching around 0.235 as the wavelength approaches 1600 nm. This trend indicates an enhancement in the interplay between the guided mode and the adjacent medium at longer wavelengths, which is a critical factor for optimizing the waveguide’s performance in sensing applications.

## 3. Characterization of RTRR

A photonic ring resonator is a compact and highly sensitive optical device widely used for RI sensing [6]. It operates by coupling light into a circular waveguide where the light resonates at specific wavelengths, which are determined by the optical path length. Changes in the RI of the surrounding medium alter the resonant wavelengths due to shifts in the n_eff_ value of the guided modes. This shift is highly sensitive to even minute changes, enabling the precise detection of biomolecules, gases, or other analytes [4]. The small footprint, compatibility with integrated photonics, and ability to provide real-time, label-free sensing make photonic ring resonators a powerful tool in optical sensing applications [5]. In this section, the fabrication process and details related to optical characterizations are discussed.

### 3.1. Fabrication Process

The fabrication of PICs involves a series of over a dozen meticulously optimized process steps [31]. In this work, all steps were conducted on 100 mm silicon wafers. The process began with a comprehensive cleaning procedure following standard protocols—RCA SC1, SC2, and Piranha treatments [32]—to remove organic and metallic contaminants from the wafer surface. After pre-cleaning, a wet thermal oxidation process generated a 2.3 μm thick SiO₂ layer, serving as an insulating barrier to separate the silicon substrate from the subsequent Si₃N₄ guiding layer. A 400 nm thick Si₃N₄ layer was then deposited using Low-Pressure Chemical Vapor Deposition (LPCVD). With the base layers in place, the designed pattern was transferred onto the wafer. Given the sub-micron scale of the features, electron beam lithography (EBL) was utilized to achieve high precision and pattern definition.

**Figure 5:**
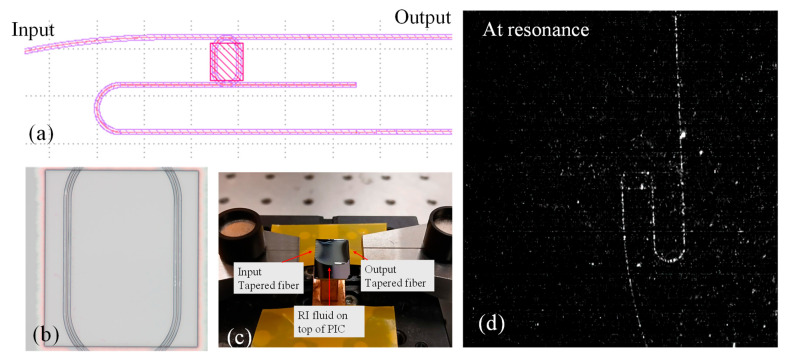
(**a**) Layout of RTRR, (**b**) microscopic image of the RTRR section, (**c**) optical characterization of the PIC, (**d**) image of the ring taken at resonance state with the help of an MIR camera.

After exposure and development, Reactive Ion Etching (RIE) was employed to transfer the pattern to the final layer, which was chosen for its anisotropic etching properties and excellent selectivity relative to the lithography resist [4]. Once RIE was complete, the remaining resist was stripped, and a 500 nm thick SiO₂ layer was deposited across the wafer using Plasma-Enhanced Chemical Vapor Deposition (PECVD). At this stage, microfluidic interfaces were integrated into the RTRRs by performing an additional photolithography step followed by localized etching of the SiO₂ layer. The fabrication process concluded with manual cleaving of the wafer to separate the individual chips. The RTRR layout is shown in Figure 5a. Figure 5b presents the microscope image of the RTRR structure, highlighting the localized removal of the SiO₂ layer. The image provides a detailed view of the precise etching and structural integrity of the racetrack, showcasing the effectiveness of the fabrication process in achieving the desired design features.

### 3.2. Optical Characterization

A broadband light source (DL-BP1-1501A SLED, Ibsen photonics, Farum, Denmark) was coupled to the bus waveguide using a tapered fiber to ensure efficient light injection. The transmitted light was collected via an output tapered fiber and directed to an optical spectrum analyzer (OSA, Yokogawa AQ6370B, Tokyo, Japan) for characterization. This configuration facilitated accurate measurement of the transmission spectrum of the RTRR. To evaluate the sensitivity of the fabricated structures, certified RI liquids from Cargille Laboratories, Cedar Grove, NJ, USA (Series AAA) were utilized [33]. Renowned for their precision and reliability, these high-quality standard liquids are extensively used in sensing and quality control applications. During the experiment, droplets of RI liquids were carefully deposited onto the sample surface, ensuring uniform coverage for accurate measurements.

After each measurement, the sample was thoroughly cleaned with isopropyl alcohol to eliminate any residual traces, maintaining the integrity of the experimental setup and ensuring repeatability. Figure 5c illustrates the PIC covered with an RI liquid selectively applied to contact only the RTRR. The PIC is positioned on a sample holder, where tapered input and output fibers are precisely aligned and coupled to opposite sides of the chip. This arrangement enables efficient light injection into the bus waveguide and collection of the transmitted light, facilitating the characterization of the device under the influence of the RI liquid. The image of the RTRR in an on-resonance state is taken with the help of an MIR camera (Xenics Xeva), which detects an enhanced light intensity in the ring as a bright spot as shown in Figure 5d.

## 4. Discussion

**Figure 6:**
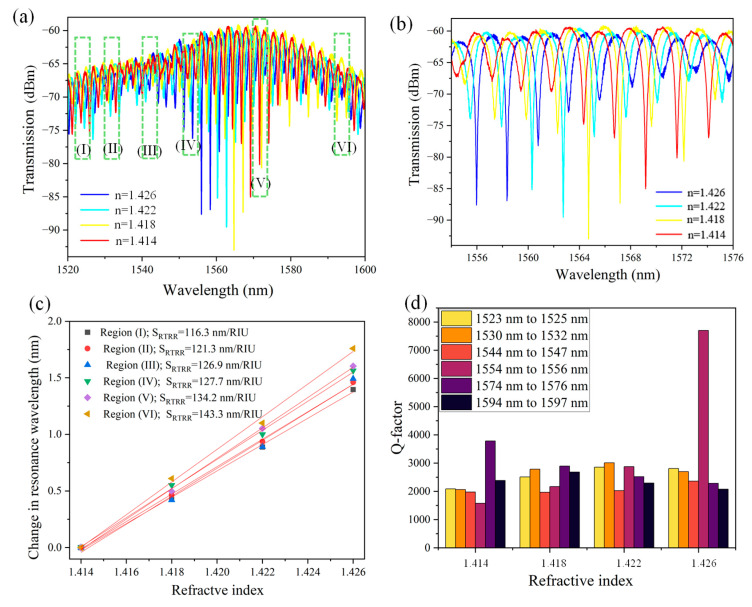
(**a**) Transmission spectrum of an RTRR in the wavelength range of 1520 nm and 1600 nm. The dotted rectangle shows the wavelength regions where sensitivity is calculated, (**b**) the transmission spectrum of an RTRR in the wavelength range IV and V, (**c**) change in resonance wavelength versus refractive index for all six wavelength ranges, (**d**) the Q-factor of RTRR.

The sensitivity of an RI sensor based on a ring resonator is commonly expressed in terms of nanometers per refractive index unit (nm/RIU) [34]. This sensitivity quantifies the shift in the resonance wavelength of the ring resonator in response to changes in the RI of the surrounding medium. The sensitivity of RTRR is calculated by utilizing Equation (5):

(5) $$S_{RTRR}=\frac{∆\lambda }{\Delta n}$$

where S_RTRR_ is the sensitivity (nm/RIU) of an RTRR, Δλ is the shift in the resonance wavelength, and Δn is the change in the RI of the surrounding medium. The transmission spectrum of the RTRR is plotted over the wavelength range of 1520 nm to 1600 nm for various RI fluids (1.414, 1.418, 1.422, and 1.426), as shown in Figure 6a. The resonance wavelength of the RTRR exhibits redshift as the RI of the ambient medium increases. This behavior can be attributed to the dependence of the resonator’s mode on the RI of the surrounding medium. As the RI of the ambient medium increases, the n_eff_ of the resonator also increases, which reduces the propagation velocity of light within the resonator. Consequently, to satisfy the resonance condition, the resonant wavelength must increase, resulting in a redshift. Moreover, Figure 6a illustrates six regions marked by green dotted boxes, which highlight the specific wavelength ranges where S_RTRR_ is calculated. These regions offer valuable insight into how the device’s sensitivity varies across different wavelengths, showcasing the wavelength-dependent nature of the response. To provide greater clarity, a magnified view of the transmission spectrum is plotted within the wavelength range of 1554 nm to 1576 nm. This detailed representation highlights the prominent resonance dips, which exhibit a redshift as the ambient RI increases, as illustrated in Figure 6b.

As observed in Figure 4b, S_w_ increases with the operational wavelength due to the enhanced EFR. To further investigate this behavior, we evaluated S_RTRR_ over six distinct wavelength ranges as indicated in Figure 6a. The S_RTRR_ is calculated to be 116.3 nm/RIU, 121.3 nm/RIU, 126.9 nm/RIU, 127.7 nm/RIU, 134.2 nm/RIU, and 143.3 nm/RIU for the respective wavelength regions of 1523–1525 nm, 1530–1532 nm, 1544–1547 nm, 1554–1556 nm, 1574–1576 nm, and 1594–1597 nm, as shown in Figure 6c. This demonstrates that the RTRR’s performance can be effectively tailored by operating the device within different wavelength regions, providing versatile control over its functionality.

The quality factor (Q-factor) of a ring resonator is a fundamental metric that characterizes the sharpness of its resonance and its ability to store optical energy [35,36]. It is defined as the ratio of the resonant wavelength to the full width at half maximum (FWHM) of the resonance dip (Equation (6)).

(6) $$Q-factor=\frac{\lambda_{res}}{FWHM}$$

where λ_res_ is the resonance wavelength and FWHM is the full width at half maximum.

A higher Q-factor indicates narrower resonance peaks, signifying lower energy losses and enhanced light confinement within the resonator. This attribute is vital for applications such as optical filtering, sensing, and nonlinear optics, where precise control over light–matter interaction is essential. The Q-factor is influenced by intrinsic material properties, waveguide geometry, coupling efficiency, and fabrication quality with higher values often requiring minimized scattering and absorption losses. The Q-factor of the RTRR is calculated for all six regions as indicated in Figure 6a for the RI values of 1.414, 1.418, 1.422, and 1.426, as shown in Figure 6d. The maximum Q-factors achieved are 7701 for region (IV) and 3784.7 for region (V), highlighting the superior resonance characteristics in these wavelength regions. A comprehensive overview of the sensor’s performance, including sensitivity, FWHM, and Q-factor values, is provided in Table 2 for detailed reference.

## 5. Concluding Remarks

In conclusion, this paper provides a comprehensive investigation into the wavelength-dependent sensitivity of a silicon nitride (Si_3_N_4_)-based ridge waveguide, combining detailed numerical analysis with experimental validation. The numerical study is conducted using the finite element method (FEM), which is a powerful computational technique that enables the precise analysis of complex structures and their behavior under various conditions. This approach allows for accurate modeling of the waveguide and its interactions with light, providing valuable insights into the performance of the system. The study begins with an in-depth examination of the modal characteristics of the Si_3_N_4_ ridge waveguide, focusing on key parameters such as the effective refractive index (n_eff_), evanescent field ratio (EFR), and propagation losses (α_prop_). These parameters are pivotal for understanding the interaction between the guided light and the surrounding medium, enabling the optimization of waveguide design for high-performance sensing applications.

The second part of the study experimentally evaluates the wavelength-dependent sensitivity of a racetrack ring resonator (RTRR) fabricated on the Si_3_N_4_ platform. The results reveal a significant enhancement in sensitivity, increasing from 116.3 nm/RIU to 143.3 nm/RIU as the operating wavelength shifts from 1520 nm to 1600 nm. Additionally, a maximum Q-factor ranging from 3784.7 to 7701 is achieved within the wavelength range of 1554 nm to 1576 nm, demonstrating the RTRR’s capability for sharp and precise resonance detection. This observed trend underscores the potential of Si_3_N_4_-based RTRRs for applications requiring high sensitivity, particularly in the longer wavelength regions. These findings not only validate the effectiveness of Si_3_N_4_ waveguide designs for advanced sensing applications but also provide valuable insights for the further development of wavelength-dependent photonic devices.

## Figures and Tables

**Table 1:** Description of the geometric parameters used in the analysis of a ridge waveguide based on Si_3_N_4_ material platform.

Variable	Description	Range
W_core_	Width of a waveguide core	800 nm to 1200 nm
H_core_	Height of a waveguide core	340 nm to 400 nm

**Table 2:** Sensitivity and Q-factor of RTRR versus wavelength range.

Wavelength range (nm)	1523–1525	1530–1532	1544–1547	1554–1556	1574–1576	1594–1597
Sensitivity (nm/RIU)	116.3	121.3	126.9	127.7	134.2	143.3
FWHM (nm)	0.534	0.508	0.654	0.2	0.415	0.59
Max Q-factor	2854.9	3011.1	2363.5	7701	3784.7	2681.8

## Data Availability

The data will be provided at a reasonable request to the author.
